# The Electronic Structure and Bonding in Some Small Molecules

**DOI:** 10.3390/molecules30051154

**Published:** 2025-03-04

**Authors:** George B. Bacskay

**Affiliations:** School of Chemistry, The University of Sydney, Sydney, NSW 2006, Australia; george.bacskay@sydney.edu.au

**Keywords:** covalent bonding, quantum chemistry, electronic structure, molecular orbital theory, valence bond theory, density functional theory

## Abstract

The electronic structures of the first- and second-row homonuclear diatomics, XeF_2_, and the weakly bound dimers of nitric oxide and nitrogen dioxide molecules in their ground states are discussed in terms of molecular orbital (MO) theory and, where possible, valence bond theories. The current work is extended and supported by restricted and unrestricted Hartree–Fock (RHF and UHF) self-consistent field (SCF), complete active space SCF (CASSCF), multi-reference configuration interaction (MRCI), coupled cluster CCSD(T), and unrestricted Kohn–Sham (UKS) density functional calculations using a polarized triple-zeta basis. The dicarbon (C_2_) molecule is especially poorly described by RHF theory, and it is argued that the current MO theories taught in most undergraduate courses should be extended in recognition of the fact that the molecule requires at least a two-configuration treatment.

## 1. Introduction

The work of Heitler and London on the quantitative description of the bonding in the H_2_ molecule using quantum theory [1] represents a revolution in the theory of chemical bonding. It inspired Pauling [2,3] and Slater [4] to generalize it for larger molecules, calling it valence bond (VB) theory. The total VB wave function, in the spirit of Heitler and London’s approach, is constructed in terms of antisymmetrized products of atom-centered orbitals (multiplied by a spin function) that represent the interaction of the H atoms, especially bonding [5,6,7,8]. The alternative technique, developed initially by Mulliken [9,10,11], Hund [12,13,14], Hückel [15], and Lennard-Jones [16], is molecular orbital (MO) theory, where the building blocks of the total wave function are antisymmetrized products of MOs, delocalized orbitals that are usually linear combinations of atomic orbitals (AOs). MO theory was initially developed as a theoretical approach to aid in spectroscopy, but with the increase in computing capacity in the 1950s and beyond, it began to be seen as a natural and simpler way to write programs for a theory that eliminated the problem of non-orthogonality and spin degeneracy. The latter issues proved to be both the advantage and disadvantage of MO-based theories over VB theory, where the non-orthogonality of the AOs (as basic building blocks) and its consequences, along with the issue of spin degeneracy, are integral aspects of the theory itself.

Most computational chemistry work today is carried out by large and efficient programs such as GAUSSIAN, MOLPRO, MOLCAS, GAMESS, etc., which are available on various platforms and can perform various quantum chemistry calculations based mostly on the MO formalism, such as RHF and UHF self-consistent field (SCF) [17,18,19,20], complete active space SCF (CASSCF) [21,22], configuration interaction (CI) [23], coupled cluster (CC) [24], and density functional theory (DFT) [25,26,27,28,29,30,31]. All DFT calculations in this work use the B3LYP exchange-correlation functional, as proposed by Lee, Yang, and Parr [32]. As discussed by Pople, Gill, and Handy [30], since a single determinant of Kohn–Sham orbitals is not the correct wave function for a given system, the apparent “spin contamination” that occurs for some open-shell systems (as if its wave function was a UHF-type single Slater determinant) is allowed. In fact, the term “contaminated” is judged as unduly derogatory [30].

Increasingly, VB theory is also part of these programs, as well as codes such as CASVB [33,34] that re-express MO-based wave functions in terms of optimized, non-orthogonal, atom-centered orbitals, as used in the full GVB approach.

Size consistency [35], e.g., the correct dissociation to atoms (that can be independently treated), and size extensivity [36] have been considered when choosing the methodology to be applied. Thus, the SCF, CASSCF, full CI, and UKS theories are size-consistent (and size-extensive), as is CCSD(T), but not multi-reference CI (MRCI). However, one needs to rely on the latter method in regions where coupled-cluster theory is inapplicable, e.g., where bond breaking occurs, i.e., where a multi-configuration treatment is necessary. The simple correction for quadruple (Q) excitations, e.g., as proposed by Davidson and others [37,38], corrects the calculated MRCI energy to a large extent. In this work, single-reference CI(SD) energy is only computed for H_2_, where it is actually full CI, i.e., size-consistent.

Basis set superposition error (BSSE) [39] is also a factor to be considered, especially in the computation of small interaction (bonding) energies. BSSE can be largely eliminated by the use of reasonably large basis sets; nevertheless, the safest way to check and account for its possible presence is by the counterpoise method of Boys and Bernardi [40]. Thus, given a complex of monomers *A* and *B*, one can correct for BSSE by recalculating the monomer *A* and *B* energies in the full *A-B* basis set.

Relativistic (Darwin, mass–velocity, and spin–orbit) effects [41], considered to be very small in comparison with electron repulsions, were ignored in this work, except in the case of XeF_2_. That decision was justified by the computations in the work of Visscher and Dyall [42], who found that molecular properties, including the equilibrium bond lengths and bond energies of the F_2_ and Cl_2_ molecules, are barely affected by explicit consideration of relativistic corrections.

Despite the fantastic increase in interest and activity in quantum chemistry during the last half-century, the great knowledge and understanding of chemical bonding has seemed to have little impact on undergraduate syllabi and texts, with a perfunctory presentation of MO theory that highlights its historic successes (such as the prediction that the oxygen molecule’s ground state is a triplet, ^3^Σ_g_^−^). The calculation of bond orders of the first-row homonuclear diatomic molecules Li_2_-F_2_ is now standard first-year material. The bond order, defined as half the excess of “bonding” electrons over “antibonding” ones, is understood as being equivalent to the number of shared pairs of electrons that, according to Lewis’s theory [43], equals the number of bonds connecting the two atoms. Thus, N_2_ has a triple bond, but C_2_ has a double bond (according to qualitative MO theory). This last result seems to go against the experimental evidence regarding bond strengths, as well as the recent arguments of Shaik et al. [44,45], based on their VB calculations, that C_2_ in fact has a quadruple bond. Xu and Dunning [46], questioning that description, proposed a different interpretation on the basis of their generalized valence bond (GVB) studies. According to them, “C_2_ is best described as having a traditional covalent σ-bond with the electrons in the remaining orbitals of the two carbon atoms antiferromagnetically coupled”.

**Figure 1:**
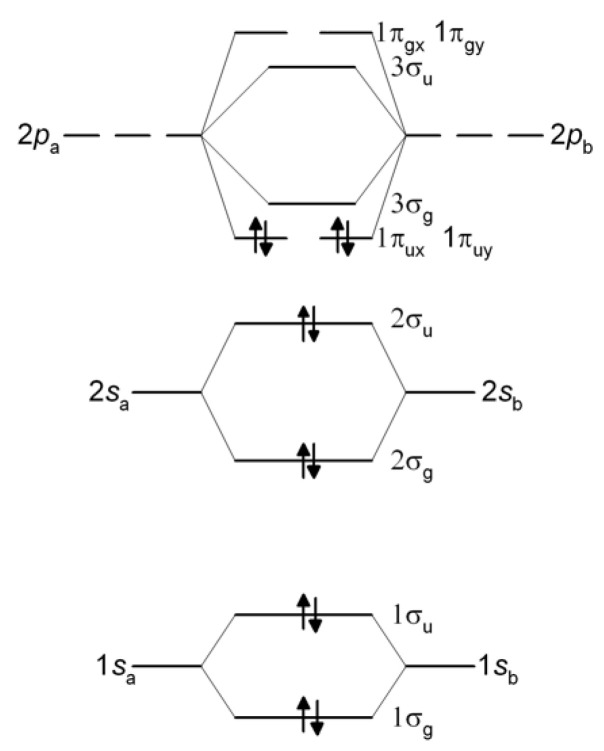
MO energy level diagram showing the “standard” configuration of C_2_.

The standard energy level diagram for C_2_, which indicates the dominant origin of the MOs, is presented in Figure 1. A straightforward generalization of the above qualitative MO view is the assumption that RHF theory adequately describes the bonding, i.e., electron correlation (static and dynamic) is unimportant. This paper looks at the latter issues and discusses the results of MO-based calculations of H_2_, the first-row diatomics Li_2_-F_2_, and other simple molecules, presenting arguments that will hopefully help resolve the differences between the VB and MO descriptions of these systems. Most of the systems in this work have been studied by a variety of methods, and the results will be familiar to many chemists. This paper brings them all together and presents the results of a variety of popular techniques using the same basis sets. As such, it should be of interest, especially to “non-experts” of quantum chemistry, and to those engaged in teaching it.

The calculation of bond strengths and potential energy curves requires one to consider electron correlation, both dynamic and static, i.e., treatments that go beyond restricted Hartree–Fock, the popular single-reference MO theory. Relaxing the restriction of doubly occupied MOs to represent the closed-shell part of a molecule, by allowing wave functions to become “unrestricted” (UHF), seems to go a long way towards resolving static correlation. On a formal level, UHF is associated with Hartree–Fock instability and the need for multi-configuration wave functions for an adequate description of an electronic state; as such, it is worth investigating [47,48,49,50,51,52,53,54,55].

## 2. Computational Methods

All of the calculations were carried out using the MOLPRO programs [56,57,58,59,60,61,62,63,64,65,66,67] on *gadi*, the Fujitsu supercomputer of the NCIS facility of Australia. The basis sets are a polarized valence triple-zeta set with core–valence correlation, aug-cc-p*w*CVTZ for the molecules with first- and second-row atoms [68,69,70], and the aug-cc-p*w*CVTZ-PP basis [71,72], in conjunction with the appropriate pseudopotential representing the inner core of the nucleus and the 28 inner-shell electrons that occupy the atomic orbitals 1*s* to 3*d* [73,74,75,76]. The H basis is just the aug-cc-pVTZ basis [69,70], while the Li and Be bases are aug-cc-pCVTZ [68] without *w*, which represents the *weighted* core–valence contribution. Using basis sets with a balanced description of the core and inner valence electrons was a consideration in the plans for the density functional calculations, since they require a good description of the overall density, including the core regions of atoms. Given that the same basis sets are used for all calculations, be they CI or coupled-cluster type, they all routinely include core and core–valence correlations.

The same type of basis (aug-cc-p*w*CVTZ) was subsequently used for the second-row homonuclear diatomics Na_2_-Cl_2_. These systems were actually treated by Peterson and Dunning [68] in their development of the cc-p*w*CV*n*Z bases (*n* = 2–5) for the second-row elements Al-Ar (as well as for the first-row elements B-Ne).

The experimental values of the dissociation energies, *D_e_*, for most systems in this work were obtained (as *D*_0_) from the Active Thermochemical Tables [77] for the atoms, to which the zero-point vibrational energies (ZPE) [78] were added, unless stated otherwise. The latter, for a diatomic system, could of course be simply calculated from the (experimental) harmonic frequency ω_e_ and the anharmonicities ω_e_*x*_e_ and ω_e_*y*_e_. The experimental bond lengths as well as the above vibrational data were taken from the tabulations of Huber and Herzberg [79]. Atomic and derived units used in this work and their relation to SI units can be found in Appendix A.

## 3. Comparison of Molecular Orbital and Valence Bond Theories

As already indicated, it is MO theory that is used and discussed in this work, yet when it comes to bonding, VB theory is an alternative, viewed by many as possibly being superior. A brief comparison of the two theories is definitely called for.

As the name implies, the basic one-electron functions in MO theory are MOs, i.e., an orthogonal set of symmetry-adapted delocalized orbitals, usually obtained by a restricted Hartree–Fock (RHF) calculation by minimizing *E*_RHF_, the energy of the full RHF wave function, which is a single Slater determinant and, by definition, uncorrelated. The correlation energy, *E*_corr_, is defined accordingly as [80]

*E*_corr_ = *E* − *E*_RHF_ (1)

where *E* is the energy from a (correlated) calculation. In light of the shortcomings of Hartree–Fock theory, correlation is further subdivided into dynamic and static (or non-dynamic) components [52,81,82]; the latter, also known as the resolution of near-degeneracy effects in molecules, is mostly associated with the phenomenon of *incorrect dissociation*, where an RHF wave function dissociates into a mixture of atoms and ions, and where the neutral atomic energies become degenerate or nearly so. The resolution of this problem is either by allowing the wave function to become multi-configurational (such as by allowing the wave function to be multi-configurational SCF or CASSCF), or by an unrestricted Hartree–Fock (UHF)-based calculation. The dynamic correlation, i.e., the electrons avoiding each other [52,81,82], is then partially resolved by further configuration interaction (CI) or by density functional theory (DFT) calculations, generally all MO-based.

By contrast, VB theory, predominantly used to explain and quantify bonding, was originally formulated in terms of AOs of the bonded atoms. Although it grew out of the Heitler–London theory of covalent bonding in H_2_, the original VB theory was semi-empirical at best. The modern formulations, known collectively as generalized VB (GVB), follow the work of Gerratt and Lipscomb [83] and of Ladner and Goddard [84]. One of its variants, spin-coupled GVB (SCGVB), is described in some detail by Dunning et al. [7,8,85]. For example, an SCGVB wave function for the N_2_ molecule (with nuclei A and B) is [8]

Ψ = Â{1*s*_A_1*s*_B_2*s*_A_2*s*_B_σ_A_σ_B_π_xA_π_xB_π_yA_π_yB_.αβ.αβ.Θ^6^_0,0_} (2)

In Equation (2) the orbitals denoted as 1*s* and 2*s* on centers A and B are doubly occupied, while the orbitals σ_A_, σ_B_, π_xA_, π_xB_, π_yA_, and π_yB_ are singly occupied. The spin function Θ^6^_0,0_ is a linear combination of the five spin functions that represent six electrons in six orbitals, as enumerated by Kotani, indicating the spin coupling of each. At around equilibrium, the dominant spin contribution is (αβ − βα)(αβ − βα)(αβ − βα), consistent with a triply bonded nitrogen molecule (with a wave function described as *perfect pairing* approximation), a straightforward generalization of the Heitler–London wave function for H_2_. At dissociation, however, the dominant spin function is αααβββ − βββααα, i.e., the spins of two nitrogen (^4^S) atoms (if the orbitals are arranged sequentially on each atom). The occupied orbitals obey the strong orthogonality requirement, i.e., intra-group non-orthogonality, but inter-group orthogonality. In our example of N_2_, the σ AOs on the two nuclei are not orthogonal, nor are the π_xA_ and π_xB_ pair of orbitals (or the π_y_ pair), which ensure correct dissociation. Also, just as in Hartree–Fock theory, at each distinct geometry, the occupied orbitals, as indicated in (2), are optimized. The major difference between the Hartree–Fock (MO) and GVB descriptions of the molecule is in the representation of ”bonds”. In the MO theory of N_2_, for example, it is an occupied bonding MO that is doubly occupied, while in (G)VB it is a Heitler–London type two-configuration wave function built from non-orthogonal atom-centered orbitals that ensures correct dissociation. Thus, the total VB wave function is intrinsically multi-configurational, i.e., correlated, while its MO counterpart, a single determinant, is uncorrelated.

## 4. Theoretical Treatments of First- and Second-Row Diatomics and Other Small Molecules

The methods used in this work are the popular coupled cluster with singles, doubles, and perturbative triples, CCSD(T), the density functional UKS(B3LYP), and the CASSCF and MRCI techniques. UHF was routinely used as a test of the RHF stability, and also as the starting point for the UKS treatments. The critical quantities calculated were the bond lengths and dissociation energies. The CCSD(T) and UKS results are summarized in Table 1, where they are compared with experimental results. In the case of the second-row diatomics Al_2_-Cl_2_, the computed bond energies (*D_e_*) from this work are compared with the spin–orbit-corrected experimental values of Peterson and Dunning [68]. 

## 5. The Hydrogen and Nitrogen Molecules, H_2_ and N_2_

**Figure 2:**
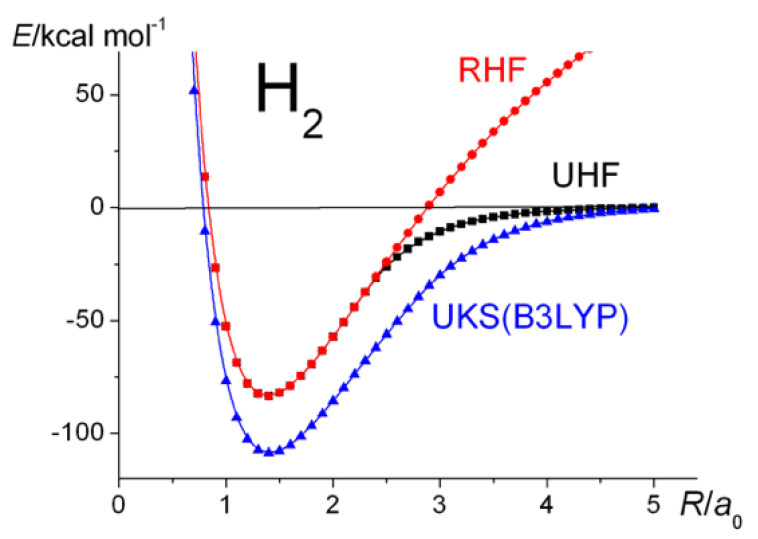
H_2_^1^Σ_g_^+^: Ground-state potential energy curves calculated at the RHF, UHF, and UKS levels of theory.

**Figure 3:**
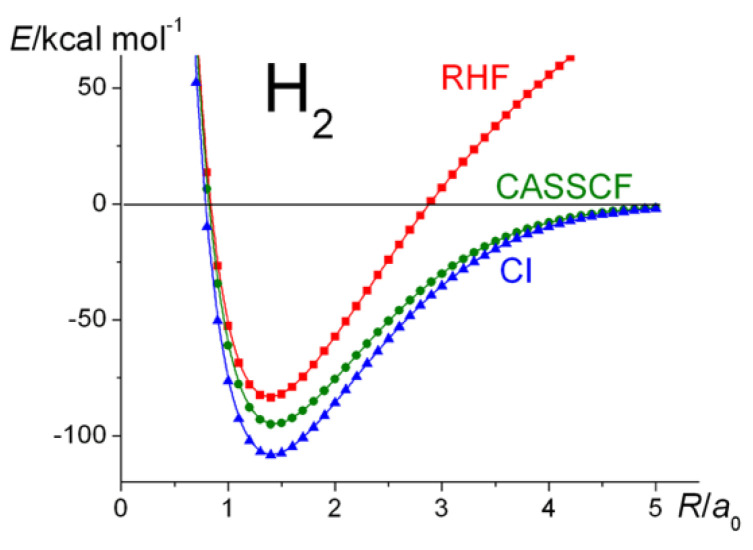
H_2_^1^Σ_g_^+^: Ground-state potential energy curves calculated at the RHF, CASSCF, and CI levels of theory.

As a benchmark, calculations, in the spirit of the larger molecules, were carried out on H_2_. The results are summarized in Figure 2 and Figure 3. The dissociation energy, *D*_e_, obtained at the Hartree–Fock level of theory, i.e., ~83.5 kcal mol^−1^, is about 77% of the UKS and CI values, which are almost identical, i.e., the correlation contribution is about 23.0% of the total. Thus, apart from the “incorrect dissociation” problem (which is present in all RHF calculations), RHF theory seems to yield a reasonable estimate of the atomization energy. The Coulson–Fischer point [47], i.e., where the bifurcation between RHF and UHF begins, is at around 2.3 a_0_. CASSCF with two active MOs yields a qualitatively correct potential energy curve and resolves ~53.4% of the correlation energy. Unfortunately, with regard to the total energies, the accuracy of the Hartree–Fock models is not maintained in the heavier molecules.

The nitrogen molecule, N_2_, is well known as a triply bonded molecule, very stable, and quite unreactive, with a dissociation energy of ~228 kcal mol^−1^. Its geometry is fairly well described at the RHF level, but in order to obtain a reasonable estimate of its dissociation energy, *D*_e_, one needs to account for electron correlation effects that are responsible for just under 50% its value. With today’s exchange-correlation functionals, such as B3LYP [32], density functional theory (DFT) is capable of describing the energetic effects of correlation very accurately. Thus, the application of unrestricted Kohn–Sham (UKS) theory [26], i.e., DFT, while comparable cost-wise with that of a Hartree–Fock calculation, yields near-perfect accuracy of energetics, including the *D_e_* of N_2_, comparable with the accuracy of multi-reference CI (MRCI) with Davidson’s correction, i.e., MRCI + Q. Given the importance of correlation, both static and dynamic [52], for the energetics of bonding, as seen here for N_2_ for example, one might also design the computational approach with correlation in mind, as with the recent work of Dunning and Xu [85], who used both VB and MO approaches in their work on the quantification of dynamic correlation effects.

**Figure 4:**
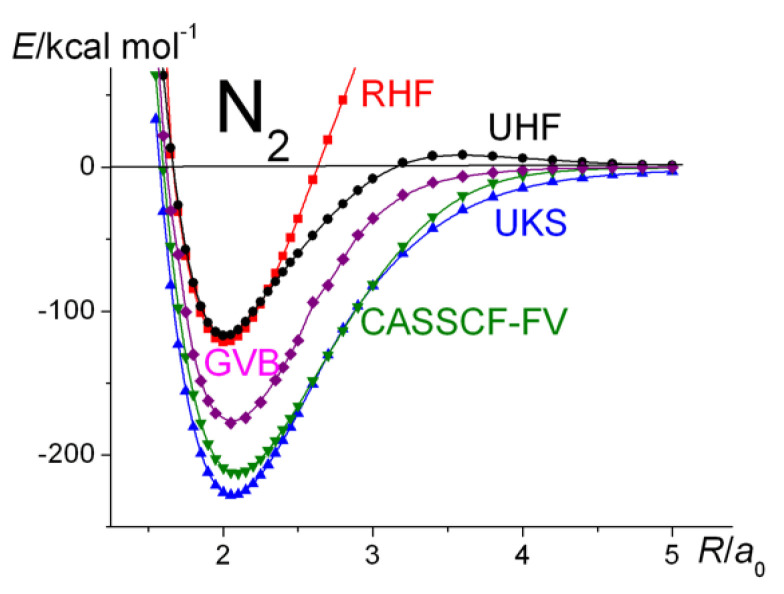
N_2_^1^Σ_g_^+^: Ground-state potential energy curves calculated at various levels of theory.

**Figure 5:**
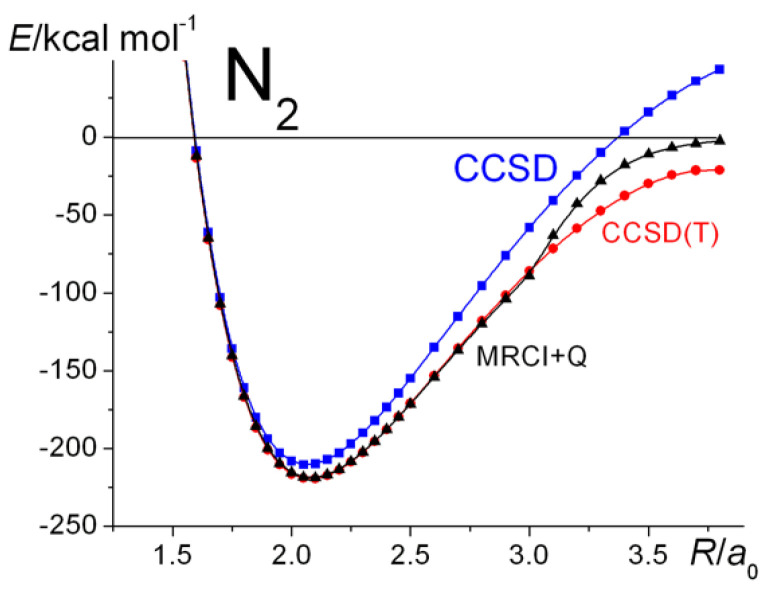
N_2_ ^1^Σ_g_^+^: Comparison of the CCSD, CCSD(T), and MRCI + Q potential energy curves.

The N_2_ results are summarized in Figure 4 and Figure 5. The UHF potential energy curve, as for H_2_, deviates from RHF at distances past ~2.2 a_0_, while the UKS curve is in good agreement with that obtained by MRCI theory, especially if the latter is ”cluster corrected” by Davidson’s method (MRCI + Q) [37,38]. The plots, actually of dissociation energies, show the UHF curve slightly higher in energy at around the equilibrium geometry, although the two theories yield exactly the same total energy. This is because the RHF and UHF energies of the atoms are slightly different. The same slight difference occurs whenever the two theories yield different energies for the atoms. Figure 4 also shows the full-valence, i.e., (10/8), CASSCF dissociation curve, which, as expected, displays essentially the same behavior. Reducing the active space to (6/6) yields essentially an identical curve to the CASSCF-FV one. The GVB curve (on the basis of the CASSCF(6/6) results, obtained by CASVIB [57,58] (shown in violet)), indicates just how well a CASSCF calculation can be reproduced by SCGVB.

The CCSD(T) results (which include, by definition, the connected triples contributions obtained perturbatively [102]), closely match those calculated by MRCI + Q at distances less than about 3.0 a_0_, i.e., the region where the wave function is dominated by the RHF determinant, as indicated by the *T*_1_ diagnostic of Lee and Taylor [103,104], that is below ~0.02 (which is also roughly indicative of the region where perturbation theory implicit in the acronym (T) in CCSD(T) is valid). To test the magnitude of BSSE in the current calculations, at the equilibrium geometry, the counterpoise correction [40] to the CCSD(T) energy was computed, finding a small error of 1.38 kcal mol^−1^, which is negligibly small in the current context.

Lewis’s theory [43] describes the bonding in N_2_ by the sharing of three electron pairs. According to qualitative VB theory [2,3,4], the σ and two π bonds are described by the constructive overlap of the *sp*-like hybrid orbitals as well as the 2*p_x_* and 2*p_y_* AOs from each atom. According to qualitative MO theory, the electronic structure consists of 3*σ_g_*, 1*π_ux_*, and 1*π_uy_* doubly occupied bonding MOs, in addition to (the doubly occupied) 1*σ_g_*, 1*σ_u_*, 2*σ_g_*, and 2*σ_u_* MO’s (with no net bonding). The formula for bond order applies nicely in this case, as well as for H_2_, but less well in the case of C_2_, somewhat poorly for O_2_ and F_2_, and not at all for Be_2_.

*BO* = (number of electrons in bonding MO’s − number of electrons in antibonding MO’s)/2 (3)

The recent work of Dunning and Xu [85,105,106,107] deals with chemical bonding and the role of dynamic correlation in the first-row molecules C_2_, N_2_, O_2_, and F_2_ (as well as N_2_, P_2_, As_2_, Cl_2_, and Br_2_). Assuming that the spin-coupled generalized valence bond (SCGVB) approach that they used accounts for the effects of non-dynamic correlation, by performing additional high-level multi-reference CI calculations, they were able to obtain good estimates of the total correlation energies and, hence, of the dynamic parts and their variations with bond length. While their studies yielded much valuable information on the effects of the two different types of correlation, unfortunately, no well-defined single mechanism has emerged. In other words, bonding of different atoms gives rise to different behaviors of dynamic correlation.

Without performing SCVGB calculations, it was not possible in the current work to separate dynamic correlation from its static counterpart. Nevertheless, it is useful to consider the variation in the (MRCI + Q) − CASSCF difference, which is entirely dynamic correlation and indicates a general increase with decreasing distance, opposite to the much larger trend of the (MRCI + Q) − RHF definition according to (1), which is dominated by the static component, as seen in the plots in Figures S1 and S2 in the Supplementary Materials. The presence of the local minimum in the plot in Figure S2 is not a characteristic feature of all diatomic molecules, as indicated by later plots for O_2_ and F_2_.

In another interesting study, Xu and Dunning [108] investigated the nature of triple bonds in the N_2_, HCN, and HCCH molecules. They concluded that the differences may well be responsible for the differences in reactivities of these molecules. 

## 6. The C_2_ Molecule

Since we all know that carbon is a tetravalent atom, the C_2_ molecule in its ground state may be expected, on the basis of Lewis’ theory [43], to be simply two C atoms connected by four bonds. As the ground state of a carbon atom is 1*s*^2^2*s*^2^2*p*^2^, the valence bond (VB) description would specify the promotion of a 2*s* electron to a 2*p* atomic orbital (AO), followed by hybridization of the 2*s* and 2*p* AOs and overlap of the resultant hybrids, creating a triply bonded molecule, with each atom having a non-bonding electron or, in fact, a quadruply bonded molecule, where the in-phase interaction of the non-bonding AOs would give rise to the weak fourth ”inverted” bond, as proposed by Shaik et al. [44,45]. However, on the basis of their GVB studies, Xu and Dunning [46] concluded that “for the internuclear distances of molecular interest, the GVB wave function of C_2_ is a mixture of the quasi-atomic configuration and the perfect pairing configuration. The complicated nature of the wave function for C_2_ provides a natural explanation for the fact that the properties of this molecule do not fit into the well-established pattern for the H_n_C_2_H_n_ series and makes clear why it is not possible to assign a definitive bond order (double, triple, quadruple) to C_2_”.

However, the near-degeneracy of the 1π_u_ and 3σ_g_ MOs raises the possibility of a triplet ^3^Π_u_ state (with a configuration …2σ_u_^2^π_u_^3^3σ_g_^1^) being the ground state. In fact, prior to the spectroscopic work of Ballik and Ramsay [109], the ground state of C_2_, based largely on the evidence of experimental observation of the Swan bands (^3^Π_g_ − ^3^Π_u_) in absorption, was believed to be ^3^Π_u_, despite the then-recent observation of the Mulliken (^1^Σ_u_ − ^1^Σ_g_) and Phillips bands (^1^Π_u_ − ^1^Σ_g_^+^) involving the lowest singlet state having also been observed in absorption. Ballik and Ramsay’s work indicated that the lowest vibrational level of the ^3^Π_u_ state lies about 610 cm^−1^ above that of the ^1^Σ_g_^+^ state, confirming the latter as the correct ground state. The results of the high-level MRCI + Q computations [110] were in fact in agreement with the above observation, placing the minimum of the ^3^Π_u_ curve 600 cm^−1^ above the minimum of the analogous ^1^Σ_g_^+^ curve. A good summary of the current experimental and theoretical determinations of the energy levels of C_2_ is provided by Furtenbacher et al. [111]. One of the earliest ab initio works was the CI study of Fougere and Nesbet in 1966 [112], which highlighted the richness of the low-lying electronic states of C_2_ and the difficulty in correctly predicting the ground state as ^1^Σ_g_^+^. The DFT calculations of Gunnarsson et al. [27], representing one of the earliest examples of such work on diatomic molecules, including C_2_, failed to actually predict the ^1^Σ_g_^+^ state to be lower in energy than the triplet state ^3^Π_u_—a problem that seems to persist to this day, as the results of Table 1 show. In the DFT study of Harris and Jones [113], the authors discuss the properties of the Group IVA dimers C_2_-Pb_2_, particularly the special nature of the C-C bond of C_2_ and its consequences in comparison with Si_2_, Sn_2_, and Pb_2_.

Simple molecular orbital (MO) theory, using Formula (3), predicts C_2_ to have a double bond, unsupported by an underlying σ-bond, due to the four electrons occupying the two bonding π MO-s (1π_u_). The remaining eight electrons occupy the two bonding (1σ_g_, 2σ_g_) and corresponding antibonding (1σ_u_, 2σ_u_) MOs. The overall bond order, as defined in Equation (3), is therefore 2. That is the result that a Hartree–Fock (HF) self-consistent field (SCF) wave function with doubly occupied 1σ_g_, 1σ_u_, 2σ_g_, 2σ_u_, 1π_ux_, and 1π_uy_ MOs yields. Such a “restricted” wave function does not dissociate correctly, as is known, i.e., in addition to the C atoms in their ^3^*P* ground state, the RHF dissociation products include a number of ions in various electronic states, in particular doubly and singly charged C ions with configurations such as C^2+^(1*s*^2^2*s*^2^) and C^2−^(1*s*^2^2*s*^2^2*p_x_*^2^2*p_y_*^2^). Allowing the wave function to become unrestricted, correct dissociation is restored, but the single-configuration UHF wave function, when different from RHF, does not correspond to a pure singlet state, i.e., the (spin) expectation value of *S*^2^ is non-zero. In molecules such as H_2_ and N_2_, UHF and RHF are identical at smaller distances than the onset of bifurcation, i.e., the RHF wave functions of those molecules are stable. In the case of C_2_, on the other hand, the RHF wave functions are unstable at *all* geometries, i.e., there are substantial differences between the UHF and RHF wave functions, as shown by the results in Figure 5. That behavior, as already noted by Tsuchimochi et al. [20], is an indication of substantial static correlation due to near-degeneracy between the RHF occupied 2σ_u_ and unoccupied 3σ_g_ MOs. The correct dissociation observed in the UHF calculations further indicates that, at large values of *R*, it is the π MOs that are responsible for the unphysical behavior of the RHF curve. Although the smallest CASSCF wave function that dissociates correctly is 6/6, in this work, all effects, including the near-degeneracy of the 2s and 2p AOs, are properly accounted for by the full-valence CASSCF calculations, i.e., eight electrons in eight active orbitals.

**Figure 6:**
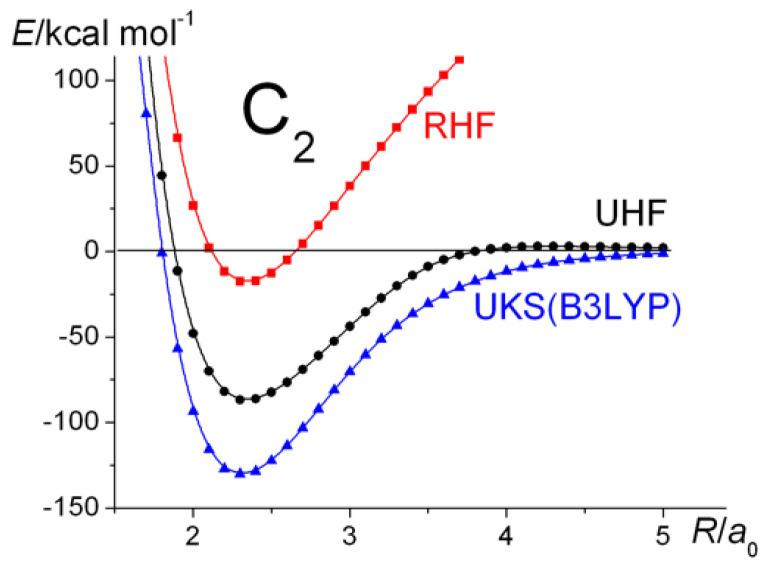
C_2_ ^1^Σ_g_^+^: Ground-state potential energy curves calculated at the RHF, UHF, and UKS levels of theory.

**Figure 7:**
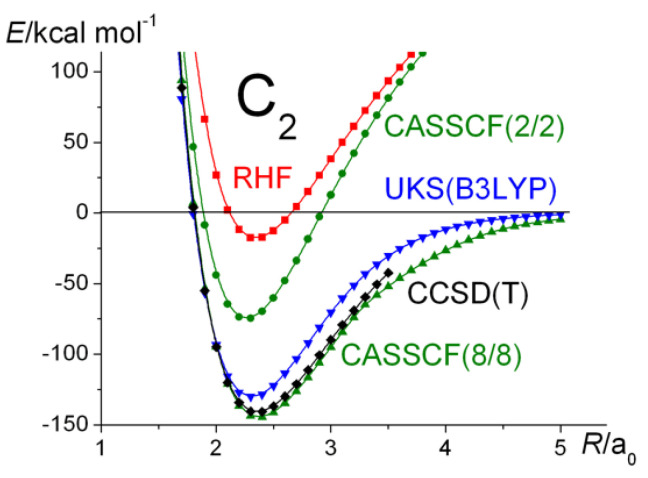
C_2_ ^1^Σ_g_^+^: Ground-state potential energy curves calculated at the RHF, CASSCF(2/2), full-valence CASSCF(8/8), UKS, and CCSD(T) levels of theory.

Although the *D_e_* values are very different, the predicted bond lengths are near-identical. The UKS results are also displayed in Figure 6 and Figure 7, while the latter shows the (2/2) and the full-valence CASSCF potential energy curves in addition to those obtained at the RHF, UKS, and CCSD(T) levels of theory. In light of the large *T*_1_ value (>0.03) at equilibrium, the applicability of the CCSD(T) method was seriously questioned by Scuseria and Lee [114], who concluded that the “method should be useful in the ab initio determination of the equilibrium structures”.

The energy of the two-configuration CASSCF wave function, where the 2σ_u_ and 3σ_g_ MOs are active, is ~0.09 *E*_h_ lower than that of the RHF one (at around the equilibrium geometries), which is a clear indication of the energetic effect of the third (and possibly fourth) bond in C_2_, as the wave function, in addition to the …2σ_g_^2^2σ_u_^2^… configuration, includes the …2σ_g_^2^3σ_g_^2^… one. The coefficients of the two configurations at around the equilibrium geometry are about 0.89 and −0.46, respectively, but change to 0.96 and −0.30 by around *R* = 3 *a*_0_.

Returning to the RHF/UHF difference, UHF is significantly lower in energy, indicating the need for a multi-configuration treatment [53,54], or a “two determinant SCF” at the least. That correlation (both static and dynamic) is crucial for the accurate description of atomization (dissociation) energy is not atypical.

As for teaching bonding, one should point out that the single-configuration treatment, i.e., the configuration 1σ_g_^2^1σ_u_^2^2σ_g_^2^2σ_u_^2^1π_u_^4^, corresponding to a bond order of 2, which has been traditional, is not nearly accurate enough. One really has to consider the possibility that, in addition to the configuration above, the 1σ_g_^2^1σ_u_^2^2σ_g_^2^3σ_g_^2^1π_u_^4^ configuration (which would yield a bond order of 4) needs to be included as well, i.e., a linear combination of the two. The resulting bond order is similarly between 2 and 4.

## 7. The Li_2_, B_2_, O_2_, and F_2_ Molecules

These molecules have 2, 6, 12, and 14 valence electrons, respectively, i.e., they all have a 4-electron core that occupies the 1*s* atomic orbitals. Thus, simple MO theory predicts Li_2_ to have a single σ-bond, i.e., a doubly occupied 2*σ*_g_ MO. The computations bear this out, as the results in Table 1 and Figure S3 indicate. The RHF/UHF bifurcation occurs at ~4.7 *a*_0_, just before the RHF and UHF equilibrium separations at ~5.3 *a*_0_ and 5.5 *a*_0_, respectively.

B_2_ is an interesting molecule. The configuration of a B atom is 1*s*^2^2*s*^2^2*p*^1^. Depending on the electronic separation between the 3σ_g_ and the 1π_u_ MOs, the ground state could be a …3σ_g_^2^ singlet, a …3σ_g_^1^1π_u_^1^ triplet (^3^Π_u_), or a …1π_ux_^1^1π_uy_^1^ triplet state, i.e., ^3^Σ_g_^−^. It is the last. B_2_ is not unlike methylene, where the singlet–triplet gap is ~9 kcal mol^−1^, i.e., about one-sixth of that in B_2_. It also resembles the C_2_ molecule inasmuch as, in both B_2_ and C_2_, the dominant configurations are the singly or doubly occupied 1π_u_ MOs, predicting the absence of a σ-bond. A definitive theoretical study and computations on B_2_ have recently been carried out by Bytautas et al. [88], obtaining a *D*_e_ value of 67.72 kcal mol^−1^ for the X^3^Σ_g_^−^ state, consistent with the values of the UKS and MRCI + Q binding energies reported in this work.

The ground state of O_2_, like that of B_2_, is a triplet state, ^3^Σ_g_^−^. It corresponds to two electrons occupying the degenerate antibonding π_g_ MOs in basic MO terminology. The simple and “correct” explanation of the paramagnetism of O_2_ has been regarded as a success of MO theory over the alternative VB method, which, on a qualitative level, indicates that O_2_ could be a singlet, with the atoms joined by a σ- and a π-bond, just as Lewis predicted on the basis of four shared electrons. Pauling, in an effort to explain the existence of a triplet, proposed the existence of two-center three-electron bonds [3]. While, on a qualitative level, much of chemistry could be described by qualitative VB theory that regards each two-electron bond as the overlap of two AOs, the more complex situations, such as even the ground state of O_2_ being triplet, need another level of theory. Hence, the proposal of two-center three-electron bonds. Ultimately, in this author’s view, what really matters is the quantitative description of the electronic states of molecules, their energies, and other properties. Whether that is achieved with VB- or MO-based theory, and whether one needs to invoke new models (and names) of bonding, is hardly worth arguing about.

Both the RHF and UHF theories provide adequate descriptions of triplet O_2_ and predict a fairly good equilibrium separation (~2.2 a_0_), although the bond lengths computed at the UKS and MRCI + Q levels of theory are predicted to be longer (~2.3 a_0_). On the other hand, the UHF energies are always lower than the RHF values by about 6–15 kcal mol^−1^, although UHF follows the RHF curve fairly well to about 2.4 a_0_, when a marked departure, i.e., a bifurcation, occurs. On the other hand, both UHF and UKS appear to lead to incorrect dissociation, not twice the energy of the triplet atoms. Such behavior was explained by Cui et al. [115], who pointed out “that UHF cannot describe the dissociation of triplet O_2_ to two triplet atoms. Instead, the proper dissociation limit is reached on the singlet and quintet curves”. As shown in Figure S6, the behavior of quintet O_2_ is according to expectations, i.e., at both the UHF and UKS levels, dissociation to triplet atoms occurs by a change in electronic state, becoming either a quintet or a singlet state.

F_2_ is a closed-shell molecule, i.e., another singlet state. It is described by qualitative MO theory as just one bonding MO, 3σ_g_, that is doubly occupied (with its antibonding partner, 3σ_u_, empty). That SCF theory predicts the ground-state energy of F_2_ to be higher than that of the atoms has been appreciated for a long time. Thus, while RHF theory does yield a curve with a minimum (hence, the equilibrium bond length of ~2.5 *a*_0_), it is the only molecule in this series where UHF theory appears to have a point of inflexion at the RHF minimum and, therefore, a bifurcation point, decreasing steadily to the separated atoms’ energy. This behavior has been well known for decades, e.g., an identical plot can be found in the work of Knowles et al. [52].

Although the inclusion of the … 3σ_u_^2^ configuration, in addition to the RHF … 3σ_g_^2^ one, i.e., a CASSCF(2:2) wave function, does yield a minimum (−16.5 kcal mol^−1^ at *R*_e_ = 2.8 *a*_0_), the full-valence CASSCF calculation predicts a slightly deeper minimum of −19.17 kcal mol^−1^ at *R*_e_ = 2.7 a_0_. However, the inclusion of dynamic correlation, as accounted for by the MRCI + Q or CCSD(T) methods, does produce a good result with *D*_e_ = 34.34 kcal mol^−1^, comparable with the CCSD(T) and B3LYP values of 36.26 and 37.41 kcal mol^−1^, respectively, and the experimental value of 38.23 kcal mol^−1^ (calculated on the basis of the heat of formation of atomic fluorine at 0 K and the zero-point energy of F_2_.)

## 8. The Be_2_ Molecule

The last first-row diatomic molecule considered here is Be_2_. Since the beryllium atom with a configuration of 1*s*^2^2*s*^2^, *s-p* near-degeneracy and, thus, hybridization is undoubtedly a factor in the possibility of bonding, it is expected to reduce the Pauli repulsion in the RHF energy and increase the amount of dynamic correlation that is ultimately responsible for the existence of Be_2_. The (relatively) high instability of the RHF wave function, manifested by an attractive UHF potential energy curve that resembles the situation in C_2_, has been discussed in detail elsewhere [116,117].

It was Jones’s DFT calculations [118] in 1979 that gave a strong indication that Be_2_ is indeed a stable molecule, despite the insistence of prominent quantum chemists that, according to all of the computational evidence, the molecular potential was repulsive. However, in 1980, using the interacting correlated fragments method, Liu and McLean [119] computed the dissociation energy (*D*_e_) of Be_2_ to be 0.10 ± 0.1 eV (2.31 kcal mol^−1^ = 807.9 cm^−1^), with an equilibrium bond length (*R*_e_) of 2.49 ± 0.2 Å (4.71 ± 0.4 a_0_). It took another four years before Bondybey and English [120] demonstrated the existence of the molecule. The latest experimental values are *D*_e_ = 2.66 kcal mol^−1^ (929.7 cm^−1^) and *R*_e_ = 4.637 *a*_0_ [87], in good agreement with the recent quantum chemical predictions [121,122]. The short Be−Be distance is also an indication that the molecule is not a van der Waals molecule but is covalently bonded due to hybridization (of the 2*s* and 2*p* AOs) as well as dynamic correlation [117]. In a recent publication, Xu and Dunning [123], based on their detailed CASSCF and SCGVB studies, discussed the nature of correlation and the importance of Pauli repulsion effects in bond formation. They concluded that, while it is dependent on electron correlation, one cannot safely determine whether the static or dynamic component is dominant.

**Figure 8:**
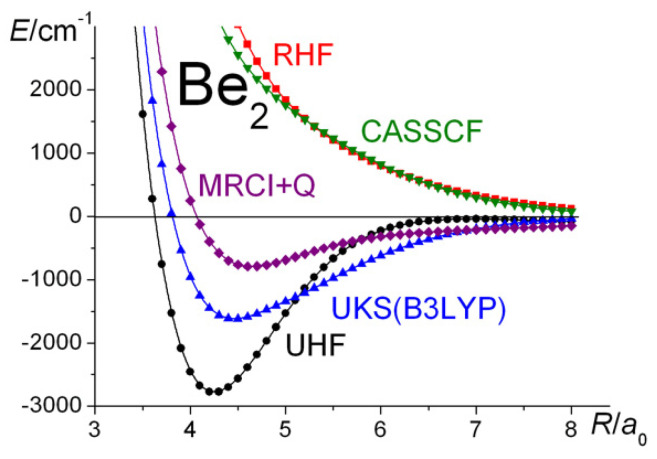
Be_2_^1^Σ_g_^+^: Ground-state potential energy curves calculated at the RHF, UHF, full-valence CASSCF, MRCI + Q, and UKS(B3LYP) levels of theory.

**Figure 9:**
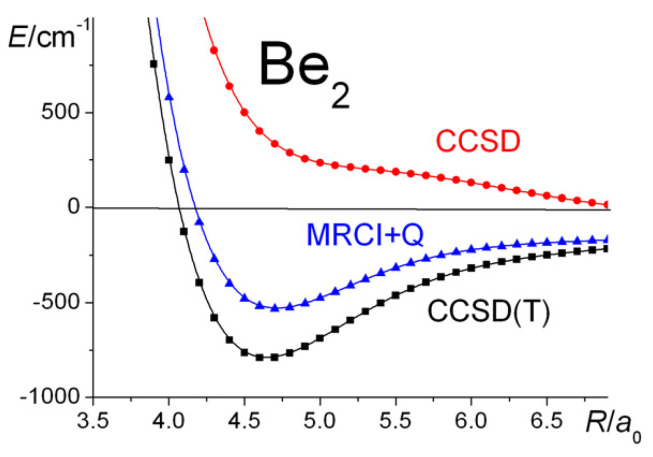
Be_2_^1^Σ_g_^+^: Ground-state potential energy curves calculated at the MRCI + Q, CCSD, and CCSD(T) levels of theory.

The current set of calculations are summarized in Table 1 and Figure 8 and Figure 9. The RHF and the full-valence CASSCF calculations, as expected, predict repulsive potential energy curves. It is only electron correlation that gives rise to an attractive potential and the existence of a molecule, i.e., only by UKS, extensive CI, or CCSD(T) calculations. Moreover, linked triple excitations are seen to be crucially important, in agreement with the conclusions of Harrison and Handy [124] on the basis of their full CI calculations. Testing the importance of BSSE in the current CCSD(T) calculations, it was found that the counterpoise corrections would decrease the dissociation energy of Be_2_ by less than 9 cm^−1^, a negligible amount in the context of this work.

## 9. Second-Row Homonuclear Diatomic Molecules Na_2_-Cl_2_

Given that the analogous set of first-row molecules are somewhat atypical, it was decided to perform a series of calculations on the second-row homonuclear diatomics Na_2_-Cl_2_, computing their all-electron dissociation energies and equilibrium bond lengths using the aug-cc-pwCVTZ basis sets at the CCSD(T) and UKS levels of theory. Peterson and Dunning [68] already performed exactly the same set of calculations for the Al_2_-Cl_2_ series, but with one difference: their basis sets did not include the diffuse functions. More importantly, however, since the differences between the ^3^Π_u_ and ^3^Σ_g_^−^ states of Al_2_ and Si_2_ were to be evaluated in the current work, the whole series was recalculated using the augmented basis sets. The results are included in Table 1, using the same “experimental” figures as those quoted by Peterson and Dunning [68].

The variations in bond lengths and dissociation from B_2_ to F_2_ compared with Al_2_ to Cl_2_ are well known and will not be discussed here apart from noting that the second-row molecules are far more uniform than their first-row counterparts. F_2_, as compared with Cl_2_ in particular, has been well documented. The ground states of both triplet-state molecules, Al_2_ and Si_2_, are correctly identified at both the CCSD(T) and UKS levels of theory.

Mg_2_, the second-row counterpart of Be_2_, a van der Waals molecule, has long been a fascinating molecule for chemists, especially because of the strengthening of the Mg-Mg bond in RMg-MgR molecules during the formation of Grignard reagents [125,126]. Both the CCSD(T) and UKS methods predict the existence of the Mg_2_ dimer.

## 10. The Four-Electron Three-Center Bonding: The XeF2 and ClF2^−^ Molecules

The XeF_2_ molecule (like F_3_^-^ and FClF^−^) represents the archetypal three-center four-electron (3c-4e) bond in electron-rich molecules that Pimental proposed in 1951 [127]. In short, bonding within these systems involves the two odd electrons in the 2*p*-orbitals from the two fluorine atoms that are connected to the central Xe (or F^−^) entity—that is, that formally a closed-shell system provides the two electrons that are needed to construct two Lewis bonds. XeF_2_ is one of the first compounds of the noble gas Xe in group 18 (or VIIIA); as such, it represented a major change in chemical thinking, inasmuch as a noble gas is not necessarily *inert*. Soon after the synthesis of XeF_2_, a number of bonding scenarios were put forward. Coulson [128], reviewing these, favored MO theory, although he did recognize that a VB wave function is simpler to interpret from a chemical point of view. The absence of a definitive numerical study at that time was a definite drawback. Almost a decade later, however, such an MO-CI study was carried out by Bagus et al. [129]. This represented a major advance in quantum chemistry, and it showed that the thermodynamic stability, like in the case of F_2_ or Be_2_, is due to electron correlation.

**Figure 10:**
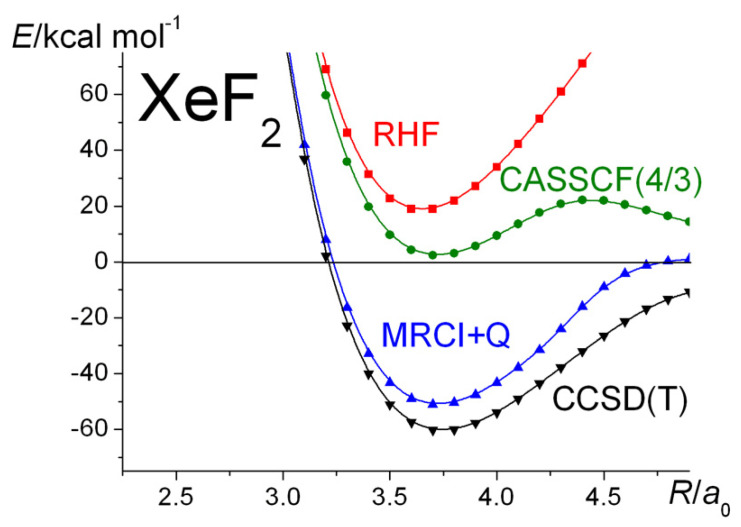
Symmetric F-Xe-F ^1^Σ_g_^+^: Ground-state potential energy curves calculated at the RHF, CASSCF(4/3), MRCI + Q, and CCSD(T) levels of theory. *R* is the Xe-F bond-length.

The results of the current calculations are summarized in Figure 10. The UHF curves, not shown in the figure, are rather like those in F_2_, i.e., at short separations, including equilibrium, the UHF energies are exactly the same as the RHF ones, but bifurcation takes place at ~4 *a*_0_, after which the UHF curve asymptotically tends to the dissociated atoms’ value. The CASSCF(4:3) curve, i.e., with three active MOs and four electrons, resembles the UHF curve, although its energy is much lower than that of the RHF one. Nevertheless, it is clear that electron correlation (implicit in an MRCI or CCSD(T) calculation) is responsible for the thermodynamic stability of XeF_2_. The experimental X-F bond length [128] and total (vibrationless) atomization energy [129] are 3.731 *a*_0_ and 66.8 kcal mol^−1^, respectively, which are in good agreement with the current CCSD(T) results.

Given the stability of XeF_2_ and the bonding scheme, it is not surprising that KrF_2_ [93], XeF_4_, and XeF_6_ also exist [95,130], as do XeF^+^, XeF, and XeF^−^ [95,130]. The compounds XeCl_2_ [131], HXeOH, and XeH_2_ [132,133] have also been identified.

In their VB studies of XeF_2_, Braïda and Hiberty [134] convincingly argued that charge-shift bonding, i.e., resonance between the covalent and ionic states, is the key feature to the bonding and stability of XeF_2_. The dominant configurations, as expected by Coulson [128], represent ion pairs, i.e., they correspond to F-Xe^+^F^−^ and F^−^Xe^+^-F.

The system ClF_2_^−^ was also studied in this work, using the same methods, basis sets, and wave functions as for the XeF_2_ molecule. The CCSD(T) and UKS results of the computed dissociation energy (relative to Cl^−^ + F + F) are given in Table 1, along with the computed equilibrium separation. The results obtained with these two methods are quite similar but, unfortunately, no experimental results seem to be available for this system.

## 11. Dimerization of NO and NO_2_

**Figure 11:**
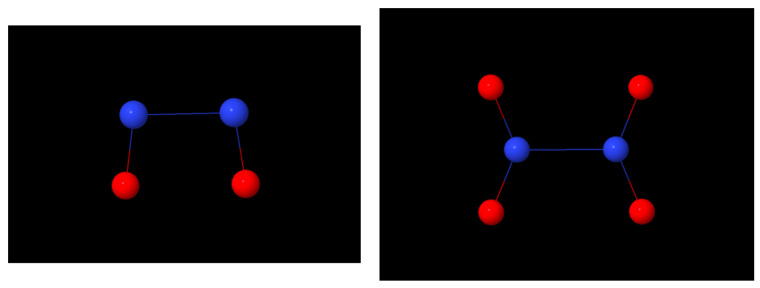
Structures of the NO (**left**) and NO_2_ (**right**) dimers.

Nitric oxide (NO) and nitrogen dioxide (NO_2_) are well-known oxides of nitrogen. Both have an unpaired electron that is largely associated with the nitrogen atom; as such, both readily dimerize (to N_2_O_2_ and N_2_O_4_, respectively), but the energetics of the dimerization processes are very different. The orbitals occupied by the odd electron are effectively a 2*p*_π_ nitrogen AO in NO, while in NO_2_ it is largely an *sp*^2^ hybrid orbital on N, as evident also from the geometry of the two dimers, as indicated in the diagrams in Figure 11. The N-N distances in the two dimers were determined to be 4.225 a_0_ [96] and 3.368 a_0_ [99], both very long, especially the former, i.e., that in the dimer of two nitric oxides. A typical N-N distance is ~2.8 a_0_. The experimental dissociation energies (*D_e_*) of N_2_O_2_ and N_2_O_4_ are 1.35 kcal mol^−1^ (1019.8 cm^−1^) [97,98] and 16.3 kcal mol^−1^ [100,101], in fair agreement with the calculated parameters listed in Table 1.

As young chemistry research students at the University of Sydney in 1986, Robert Penfold and myself undertook a CASSCF study of the nitric oxide dimer, with the aim of determining its equilibrium geometry and dissociation energy. Unfortunately, we did not realize at the time that without accounting for a substantial amount of dynamic correlation, i.e., using CASSCF alone, the stability of the dimer could not be demonstrated. It was eight years later that Roos and co-workers [98] were able to perform the necessary MRCI-type calculations that provided a quantitative prediction of the dimer’s equilibrium geometry and dissociation energy. The current work, using the (single-reference) coupled-cluster technique, although less accurate than the calculations of González-Luque, Merchán, and Roos [98], is able to provide proof of the stability of the NO dimer and a confirmation that dynamic correlation plays a critical role in its existence.

Although the dimer of nitrogen dioxide has been known since the early 1960s [135] or even before, it was only in the 1990s that accurate calculations of the dimer became possible, although in 1983 the (18/12) CASSCF study of Bauschlicher et al. [136] was able to explain the geometry of the dimer, particularly its planarity and the long N-N distance. In 1997, Weselowski et al. [137] concluded that “high level single reference wavefunctions are good enough”, having used the CCSD(T) method with a variety of extensive basis sets to confirm the structure and the dissociation energy of the NO_2_-NO_2_ dimer, while in 2003 Ivanic [138] used a number of different versions of the occupation-restricted multiple-active-space (ORMAS) method to determine the geometry and energetics of N_2_O_4_. The current CCSD(T) results closely agree with those of Weselowski et al. [137] and of Ivanic [138], while the UKS predictions of the structure are essentially the same as those by Kovács et al. [139]

Returning to the original conundrum, why is there such a difference between NO and NO_2_? The answer may well be very simple: differences in the electron density and, consequently, the different Pauli repulsions experienced by the oncoming molecule. A simple way to model this is to consider the sideways and head-on approaches of neon or argon atoms to NO and NO_2_ respectively, calculating the interaction energy at just the SCF level. Such calculations indeed result in a greater repulsion for NO (at the same nitrogen-rare gas distance). Thus, the attractive interaction with NO or NO_2_ is reduced to a greater degree in the former, as observed.

## 12. The Rare Gas Dimers He_2_, Ne_2_, Ar_2_…

The rare gas atoms, as discussed previously [140], all have a closed-shell structure, i.e., with the exception of He, which has a 1*s*^2^ configuration, the outermost n*p* (n = 2, 3, 4, …) AOs (as well as those of lower energy) are fully occupied. Thus, no covalently bonded dimers exist (unlike the case of beryllium, where a degree of near-degeneracy between the 2*s* and 2*p* AOs is present). The Pauli repulsion between electrons of the same spin is manifested in an increase in the kinetic energy of the system and, thus, the overall repulsive potential, i.e., antibonding, which can be well estimated via an RHF calculation. The small, attractive minima [141] (at distances of 5.62, 5.86, and 7.15 *a*_0_ for He_2_, Ne_2_, and Ar_2_, respectively, with corresponding depths, i.e., *D_e_* values of 7.40, 28.40, and 96.50 cm^−1^) in the potential energy curves are due to dynamic correlation, the main source of dispersion.

## 13. Conclusions

The current valence-correlated calculations, as well as others in the literature, indicate that RHF theory is insufficient for the correct description of bonding, especially in the cases of Be_2_ and C_2_, as indicated by the large difference between the UHF and RHF potential energy curves. While both the RHF and UHF theories yield reasonable bond lengths, with the exception of H_2_, the description of correlation, both static and dynamic, is crucial for obtaining reasonable values of the dissociation energies. This necessitates the use of configuration interaction-based theories or UKS, which are able to resolve correlation’s contributions to the bonding energies of molecules. An important conclusion is, in fact, that UKS does predict bonding, i.e., equilibrium bond lengths and binding energies that are very close to those experimentally observed. While not actually used in this work, SCGVB theories provide an alternative to the MO-based approaches, especially in the description of bonding. As this work and that of Dunning and Xu (and others) [7,8,46,85,105,106,107,108,123] illustrate, the relationship between these two seemingly different theories is quite close. As a closing sentence, it is fitting to quote the title of a paper by prominent VB workers [142]: “Valence Bond and Molecular Orbital: Two Powerful Theories that Nicely Complement One Another”.

## Figures and Tables

**Table 1:** Summary of the CCSD(T) and UKS bond lengths and dissociation energies.

Molecule		CCSD(T)		UKS		Experiment ^a,b^	
	State	*R_e_*/*a*_0_	*D_e_*/kcal mol^−1^	*R_e_*/*a*_0_	*D_e_*/kcal mol^−1^	*R_e_*/*a*_0_	*D_e_*/kcal mol^−1^
H_2_	^1^Σ_g_^+^	1.404	108.55	1.404	110.14	1.401	109.50
Li_2_	^1^Σ_g_^+^	5.060	24.12	5.109	20.82	5.0510	24.59 ^c^
Be_2_	^1^Σ_g_^+^	4.721	532.5 (/cm^−1^)	4.458	1619.5 (/cm^−1^)	4.637 ^d^	929.7 ^d^ (/cm^−1^)
B_2_	^3^Σ_g_^−^	3.008	63.95	3.047	60.04	3.005	67.72 ^e^
C_2_	^1^Σ_g_^+^	2.354	141.23	2.307	130.59	2.345	146.74
C_2_	^3^Π_u_	2.484	139.41	2.459	141.96	2.491	144.42
N_2_	^1^Σ_g_^+^	2.079	219.56	2.061	228.06	2.074	228.30
O_2_	^3^Σ_g_^−^	2.288	119.1	2.278	123.49	2.282	120.25
F_2_	^1^Σ_g_^+^	2.678	36.26	2.640	37.42	2.668	38.23
Na_2_	^1^Σ_g_^+^	5.824	17.33	5.771	16.89	5.818	18.19 ^f^
Mg_2_	^1^Σ_g_^+^	7.456	61.7 (/cm^−1^)	7.455	102.3 (/cm^−1^)	7.351 ^g^	714.5 ^f,g^ (/cm^−1^)
Al_2_	*X*^3^Π_u_	5.130	32.04	5.212	30.91	5.104	31.7 ^b^
Al_2_	^3^Σ_g_^−^	4.687	30.87	4.736	28.09		
Si_2_	*X*^3^Σ_g_^−^	4.261	72.30	4.286	74.36	4.2443	75.6 ^b^
Si_2_	^3^Π_u_	4.095	70.82	4.084	70.58		
P_2_	^1^Σ_g_^+^	3.595	108.38	3.570	114.96	3.578	117.2 ^b^
S_2_	^3^Σ_g_^−^	3.589	97.54	3.596	103.03	3.570	102.9 ^b^
Cl_2_	^1^Σ_g_^+^	3.786	55.24	3.802	55.47	3.757	59.7 ^b^
ClF	^1^Σ^+^	3.090	59.45	3.098	60.75	3.077	60.39
XeF_2_	^1^Σ_g_^+^	3.742	60.38	3.785	65.68	3.731 ^h^	67.65 ^i^
ClF_2_^−^	^1^Σ_g_^+^	3.502	42.39	3.483	36.04		
ON-NO	^1^Σ_g_^+^	4.007	268.6 (/cm^−1^)	3.720	1032.7 (/cm^−1^)	4.225 ^j^	1019.8 ^k^ (/cm^−1^)
O_2_N-NO_2_	^1^Σ_g_^+^	3.303	16.25	3.408	13.30	3.368 ^l^	16.3 ^m^

^a^ Unless stated otherwise, the *D_e_* values were obtained from ATcT [77] and ZPE [78] (or from frequencies and anharmonicities from [79]). All bond lengths of diatomics were obtained from [79]. ^b^ From tabulations of Peterson and Dunning [68]. ^c^ Refs. [79,86]. ^d^ Refs. [79,87]. ^e^ Ref. [88]. ^f^ Refs. [79,89], with atomic spin–orbit coupling correction [90,91]. ^g^ Ref. [92]. ^h^ Ref. [93]. ^i^ Ref. [94] corrected for ZPE, scalar relativity, and spin–orbit coupling from [95]. ^j^ Ref. [96]. ^k^ Refs. [97,98]. ^l^ Ref. [99]. ^m^ Ref. [100] corrected for ZPE [101].

## Supplementary Materials

The following supporting information can be downloaded at https://www.mdpi.com/article/10.3390/molecules30051154/s1: Figure S1: N_2_: Energy differences between the MRCI + Q state and the RHF and CASSCF states; Figure S2: N_2_: Energy differences between the MRCI + Q and the CASSCF states; Figure S3: Li_2_^1^Σ_g_^+^: Ground state potential energy curves calculated at the RHF, UHF, UKS and MRCI + Q levels of theory; Figure S4: B_2_: ^3^Σ_g_^−^ ground state potential energy curves calculated at the RHF, UHF and UKS levels of theory; Figure S5: B_2_: ^3^Σ_g_^−^ ground state potential energy curves calculated at the RHF, CASSCF, MRCI+Q and CCSD(T) levels of theory; Figure S6: O_2_^3^Σ_g_^−^ ground state potential energy curves calculated at the RHF, UHF and UKS levels of theory; Figure S7: O_2_^3^Σ_g_^−^ ground state potential energy curves calculated at the RHF, full valence CASSCF and MRCI + Q levels of theory; Figure S8: O_2_^3^Σ_g_^−^ ground state potential energy curves calculated at CCSD and CCSD(T) levels of theory and comparison with MRCI + Q results; Figure S9: O_2_: Energy differences between the MRCI + Q and the CASSCF states; Figure S10: F_2_^1^Σ_g_^+^: Ground state potential energy curves calculated at the RHF, UHF and UKS levels of theory; Figure S11: F_2_^1^Σ_g_^+^: Ground state potential energy curves calculated at the RHF, full valence CASSCF and MRCI+Q levels of theory; Figure S12: F_2_^1^Σ_g_^+^: ground state potential energy curves calculated at CCSD and CCSD(T) levels of theory and comparison with MRCI + Q results; Figure S13: F_2_: Energy differences between the MRCI + Q and the CASSCF states; Figure S14: Cl_2_^1^Σ_g_^+^: Ground state potential energy curves calculated at the RHF, UHF and UKS levels of theory; Figure S15:Cl_2_^1^Σ_g_^+^: Ground state potential energy curves calculated at the full valence CASSCF, MRCI + Q, CCSD and CCSD(T) levels of theory.

## Appendix A

Atomic and derived units used in this work, and their relation to SI units [143]:

	**Atomic or Derived Unit**	**Relation to SI**
*length, l*	bohr, *a*_0_	$=4\pi \varepsilon_{0}ћ/m_{e}e^{2}\approx 5.29177\times 10^{-11}\;m$
*energy, U*	hartree, *E*_h_	$={ћ}^{2}/m_{e}a_{0}^{2}\approx 4.35975\times 10^{-18}\;J$
*energy, U*	kcal mol^−1^	≈6.947700 × 10^−21^ J
*energy, U*	cm^−1^	≈1.986447 × 10^−23^ J
