# Supplementary material for: The Electronic Structure and Bonding in Some Small Molecules
Source: Molecules. 2025 Mar 4;30(5):1154. doi: 10.3390/molecules30051154 (PMC11902227; doi:10.3390/molecules30051154)
Supplement: Supplementary file 1 [file molecules-30-01154-s001.zip › molecules-3425616-supplementary.pdf]

Supporting Information

The Electronic Structure and Bonding in Some Small Molecules

George B Bacskay

School of Chemistry, The University of Sydney, NSW 2006, Australia

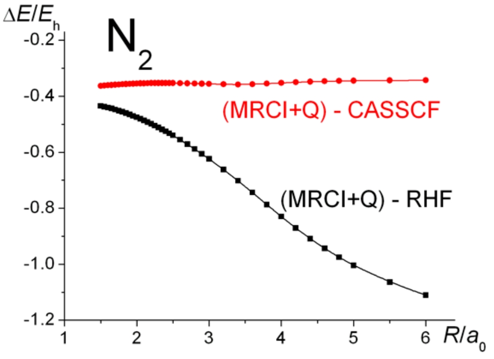

Figure S1. N_2: Energy differences between the MRCI+Q state and the RHF and CASSCF states.

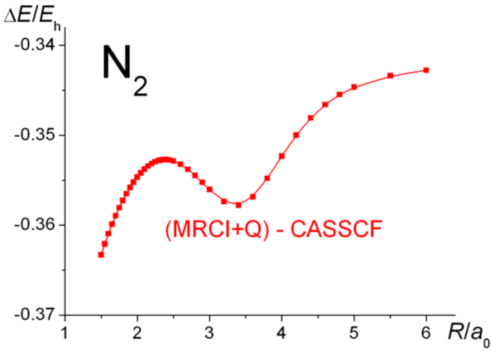

Figure S2. N_2: Energy differences between the MRCI+Q and the CASSCF states.

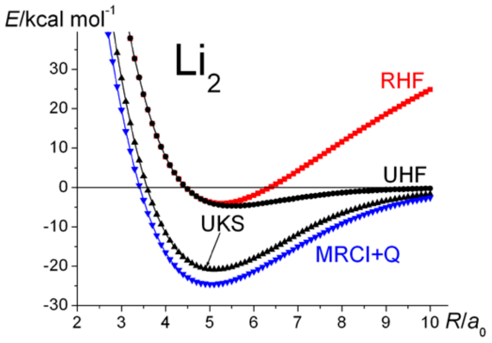

Figure S3. Li_2 $^1\Sigma_g^+$: Ground state potential energy curves calculated at the RHF, UHF, UKS and MRCI+Q levels of theory.

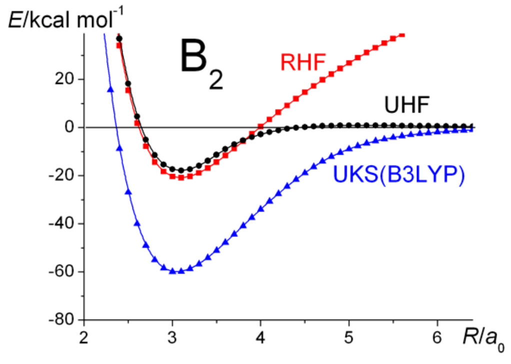

Figure S4. B_2: $^3\Sigma_g^-$ ground state potential energy curves calculated at the RHF, UHF and UKS levels of theory.

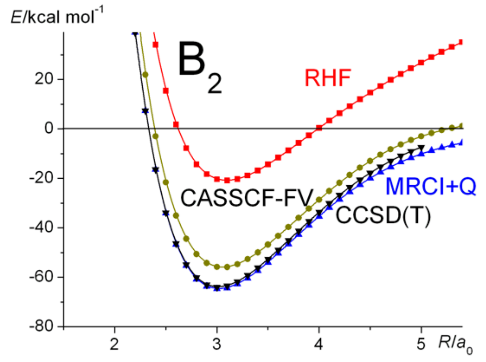

Figure S5. B_2: $^3\Sigma_g^-$ ground state potential energy curves calculated at the RHF, CASSCF, MRCI+Q and CCSD(T) levels of theory.

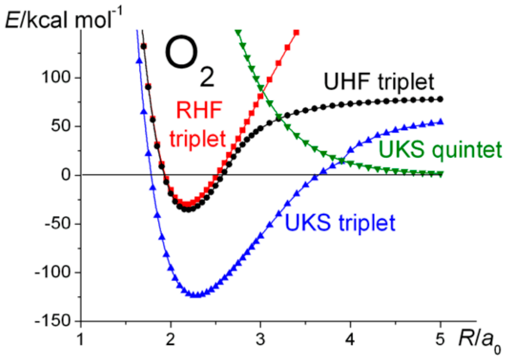

Figure S6: O_2 $^3\Sigma_g^-$ ground state potential energy curves calculated at the RHF, UHF and UKS levels of theory.

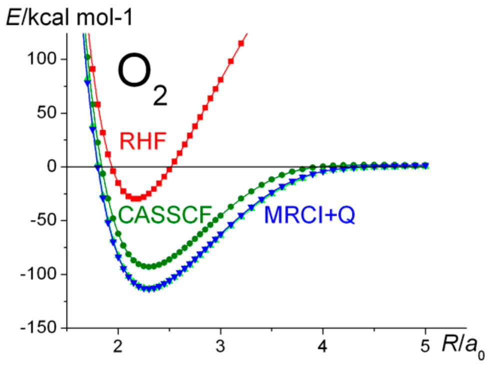

Figure S7. O_2 $^3\Sigma_g^-$ ground state potential energy curves calculated at the RHF, full valence CASSCF and MRCI+Q levels of theory.

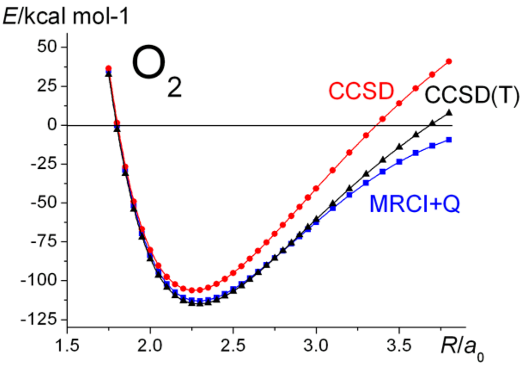

Figure S8. O_2 $^3\Sigma_g^-$ ground state potential energy curves calculated at CCSD and CCSD(T) levels of theory and comparison with MRCI+Q results.

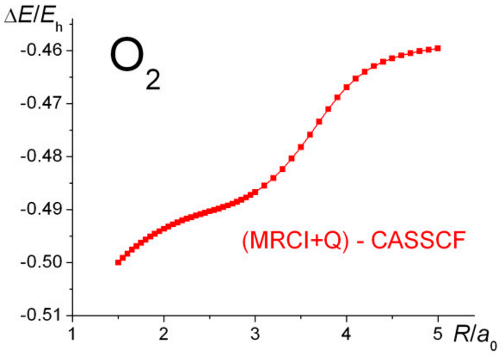

Figure S9. O_2: Energy differences between the MRCI+Q and the CASSCF states.

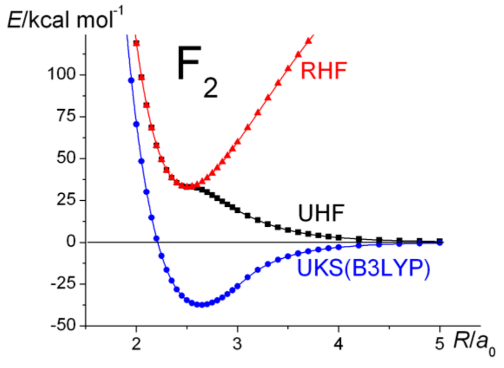

Figure S10. F_2 $^1\Sigma_g^+$: Ground state potential energy curves calculated at the RHF, UHF and UKS levels of theory.

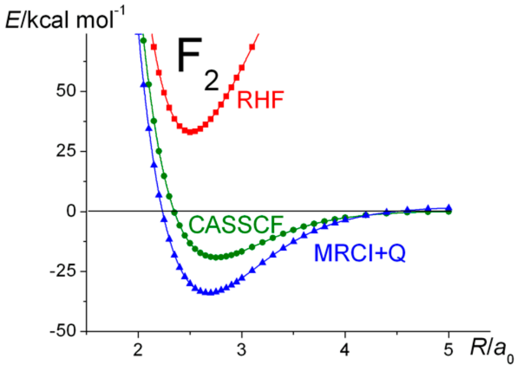

Figure S11. F_2 $^1\Sigma_g^+$: Ground state potential energy curves calculated at the RHF, full valence CASSCF and MRCI+Q levels of theory.

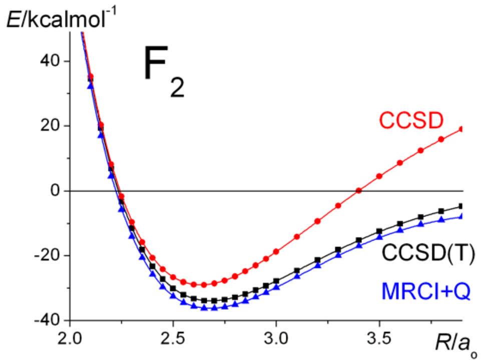

Figure S12. F_2 $^1\Sigma_g^+$: ground state potential energy curves calculated at CCSD and CCSD(T) levels of theory and comparison with MRCI+Q results.

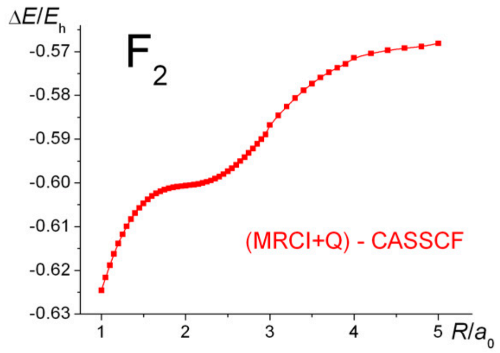

Figure S13. F_2: Energy differences between the MRCI+Q and the CASSCF states.

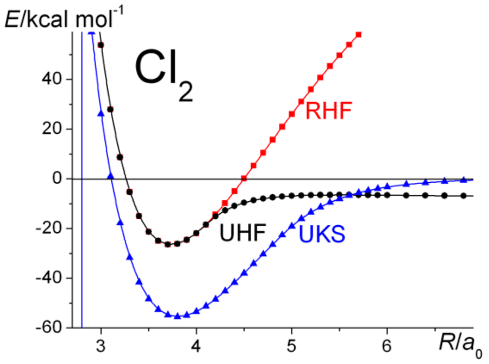

Figure S14. Cl_2 $^1\Sigma_g^+$: Ground state potential energy curves calculated at the RHF, UHF and UKS levels of theory.

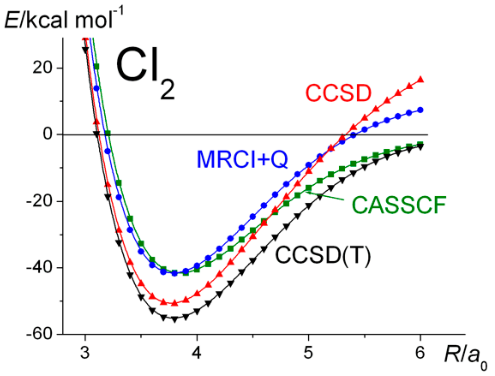

Figure S15. Cl_2 $^1\Sigma_g^+$: Ground state potential energy curves calculated at the full valence CASSCF, MRCI+Q, CCSD and CCSD(T) levels of theory.
